# Integrated multicomponent interventions to support healthy aging of the whole person

**DOI:** 10.1111/acel.14001

**Published:** 2023-10-15

**Authors:** Helene M. Langevin, Wendy Weber, Wen Chen

**Affiliations:** ^1^ National Center for Complementary and Integrative Health (NCCIH), National Institutes of Health Bethesda Maryland USA

**Keywords:** complementary therapies, healthy aging, integrated care, integrative health, integrative medicine, resilience, whole person health

## Abstract

Healthy aging is an integrated “whole person” process that involves an individual's biology, behavior, and social/physical environment. With the recent development of antiaging drugs, careful consideration of the respective roles of pharmacologic and nonpharmacologic approaches to both health and aging is in order. Recent advances in understanding the cellular and molecular mechanisms of aging are providing new measures that can be used as clinical outcomes in studying the impact of antiaging interventions in humans. This paper outlines the strategic interest of the National Center for Complementary and Integrative Health (NCCIH) in supporting the development, testing, and implementation of effective, scalable, and integrated multicomponent interventions to support healthy aging of the whole person.

## Graphical abstract

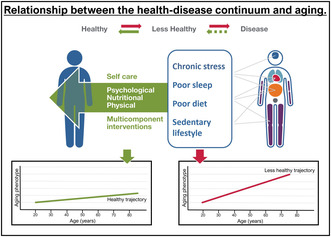

## 1 INTRODUCTION

Healthy aging is defined as the preservation of functional ability and well‐being with older age (World Health Organization, 2020). It is well established that a supportive environment and a healthy lifestyle protect against age‐associated chronic diseases and promote healthy aging (Buettner & Skemp, 2016; Nyberg et al., 2009). Although this general “prescription” has been evident for some time, it is difficult to implement due to a combination of “systemic” social and environmental obstacles to health, especially in underserved populations (National Research Council, 2013), and individual‐level challenges of sustaining behavior change over a long period of time (Bouton, 2014). Partly because of this, the biomedical response to disease prevention has predominantly relied on early detection and drugs to avoid long‐term complications from chronic conditions such as hypertension and hyperlipidemia, and now on developing drugs for managing the aging process itself. Unfortunately, the preventive pharmacologic approach to medically “controlling” specific physiological processes, such as lipid metabolism, can come at the cost of collateral effects in other organs and systems (Mansi et al., 2021). A pharmacologic approach to managing aging, although superficially attractive, may suffer similar pitfalls. Pharmacologic agents have shown promise in manipulating cellular processes associated with aging, such as cellular senescence and senescence‐associated secretory phenotypic transformation of cells. However, these pathways are also involved in the intrinsic defense against neoplasia (Carpenter et al., 2021), and it is unclear at present whether preventively manipulating them to retard aging could result in unintended effects in the long term. Translational research applying the rapidly advancing basic science of aging to the whole person provides the best opportunity to determine if these approaches safely promote healthy aging.

In this commentary, we outline the strategic interest of the National Center for Complementary and Integrative Health (NCCIH) in supporting the study of healthy aging, emphasizing rigorous research on multicomponent interventions to address the whole person. The mission of NCCIH is to study the safety and usefulness of complementary and integrative health approaches or interventions. Complementary approaches include therapies, practices, and systems with nutritional, physical, and/or psychological therapeutic inputs that may have originated outside of conventional medicine and are gradually being integrated into conventional care. Multicomponent interventions often combine two or more therapies, including conventional, complementary, or both. We argue that promoting health and healthy aging are integrated processes that are not compartmentalized but rather involve every organ and system of the body, as well as an individual's behavior and social/physical environment. In contrast, treating disease once it occurs should be precisely targeted to correct specific abnormalities. Integrating the goals of promoting health and treating disease ideally consists of a multipronged approach that collectively promotes whole‐person health, while each component can serve a distinct health‐promoting goal or therapeutic target. One example of a multicomponent intervention for healthy aging may be lifestyle interventions, such as diet, exercise, and stress management, with adequate support as primary prevention starting as early as possible, while providing medications as needed for individuals with risk factors for whom lifestyle modification is not sufficient. Importantly, we need research to provide evidence to support this combined goal. This paper outlines strategies to develop, test, and implement effective, scalable, and integrated interventions to support healthy aging in the whole person.

## 2 RELATIONSHIP OF HEALTH, RESILIENCE, LIFESTYLE, AND AGING

**FIGURE 1:**
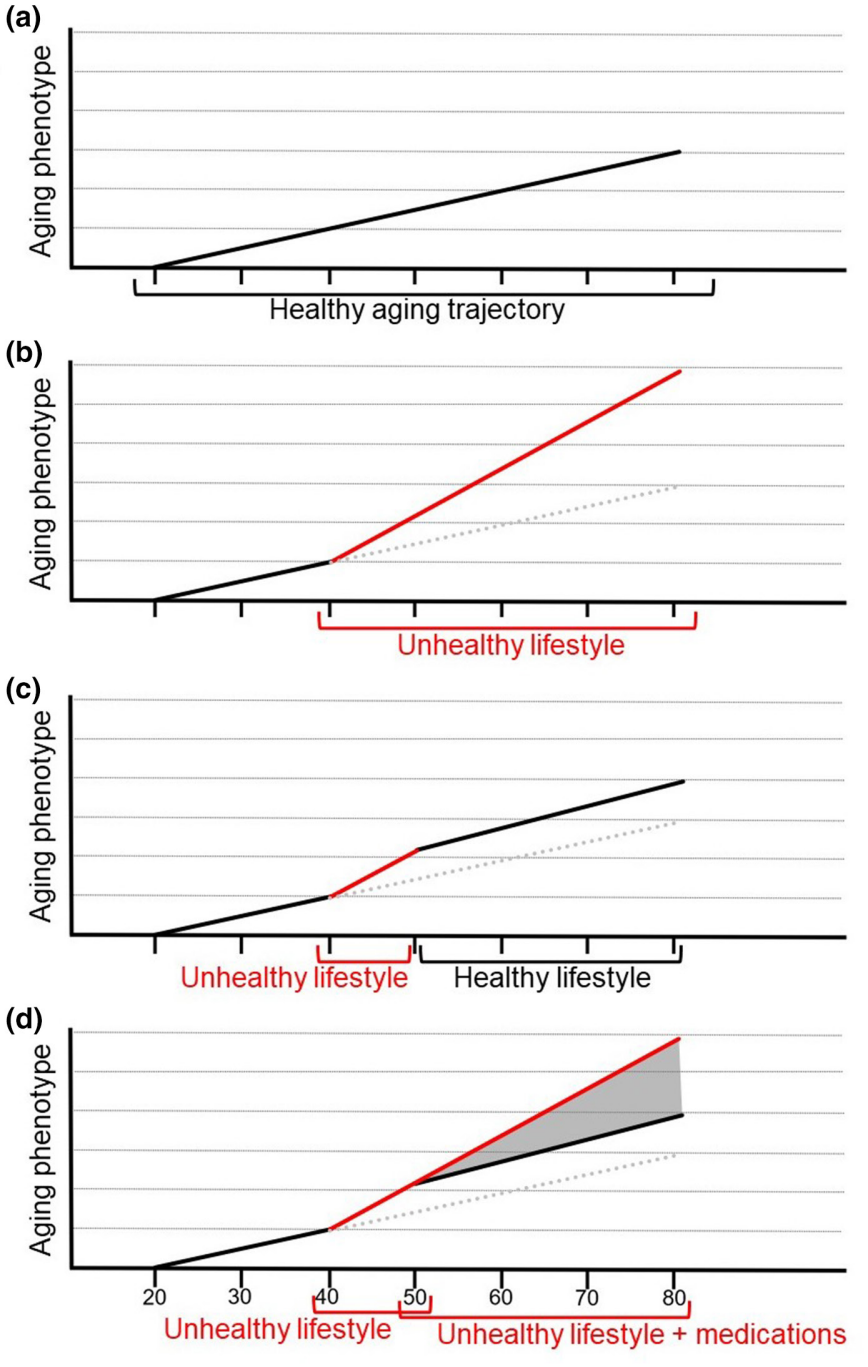
Hypothetical plots comparing (a) healthy phenotypic aging over time, (b) “bending” of the aging slope with an unhealthy lifestyle starting at age 40, (c) return to the healthy aging slope at age 50 with an improved lifestyle, and (d) range of aging trajectories that may result when medications effectively correct the biological consequences of an unhealthy lifestyle but without actual lifestyle modification. It is unclear at present whether, in such a case, the slope of the aging trajectory for such an individual would be closer to the black or the red line (slopes of the healthy and unhealthy lifestyles, respectively).

Staying healthy over a lifetime means having the ability to return to a healthy state after an accident, illness, or stressful life event—a hallmark of resilience—rather than accumulating compensatory metabolic processes and increasing the detrimental “allostatic load” throughout one's life (Maestripieri & Hoffman, 2011; McEwen, 2007). Thus, one's health “trajectory” can be bidirectional—moving away from or back toward health—as we navigate life's challenges. Aging, on the other hand, is a chronologically unidirectional process that, while it cannot be truly “reversed,” can progress at a variable rate or “pace”—that is, slow down or speed up—over the course of one's life (Belsky et al., 2020; Kuo et al., 2022). Markers of inflammation, oxidative stress, DNA methylation, and a number of composite measures incorporating clinical (e.g., blood pressure, HbA1c) and functional (e.g., grip strength, gait speed, memory) measurements have been evaluated for their ability to track the pace of aging over time (Dugan et al., 2023; Kuo et al., 2022; Lara et al., 2015). Data from longitudinal studies and small pilot trials using these markers suggest that, in a given individual, lifestyle factors can “bend” the aging curve, such that the pace of aging may accelerate or decelerate in response to, for example, gaining weight or starting an exercise program (Fitzgerald et al., 2021; Quach et al., 2017) (Figure 1a–c).

However, rigorously testing this hypothesis is not straightforward. This is because of the challenge inherent in understanding the respective impacts of lifestyle and medications used to manage chronic conditions. For example, common medications such as statins and antihypertensives can normalize some biological markers (e.g., serum lipids, blood pressure) and prevent cardiovascular events, but it is unclear at present whether they can also retard aging without concomitant lifestyle modification (Figure 1d). Large trials that randomize individuals to a multicomponent lifestyle intervention (e.g., including nutrition, physical activity, and stress management) versus continuing their current lifestyle while tracking medications would be needed to determine if a comprehensive healthy lifestyle program can slow the pace of aging, with or without medication. Selecting the optimal combination of markers to include in these studies will be key and should ideally include molecular makers of aging, such as DNA methylation, functional measures, and disease‐specific markers such as serum lipids or HbA1c, as appropriate to the patient population being studied.

## 3 WHOLE‐PERSON HEALTH AND ITS RELEVANCE TO AGING

Unlike other NIH institutes and centers that focus on a specific body system, process, or disease, the mission of NCCIH encompasses the study of treatments and practices aimed at promoting and restoring health. In its current strategic plan, NCCIH operationally defines whole‐person health as empowering individuals, families, communities, and populations to improve their health in multiple interconnected domains: biological, behavioral, social, and environmental (National Center for Complementary and Integrative Health, 2021a). Diet and physical activity are the most common factors that have been studied in relation to healthy aging and chronic disease prevention, but sleep and stress management, while often overlooked, are no less essential. In fact, chronic stress and poor sleep can be the “straw that breaks the camel's back” for individuals struggling with maintaining a healthy lifestyle (Geiker et al., 2018). Understanding this is key, because difficulty with long‐term adherence is the biggest obstacle in prevention programs based on behavior change. Furthermore, chronic stress and poor sleep, on their own, are emerging as major drivers of systemic inflammation, oxidative stress, and autonomic and immune dysregulation (Epel et al., 2018; Garbarino et al., 2021; Liu et al., 2017). When combined with a poor diet, weight gain, and insufficient physical activity, this creates a “recipe” for the most common chronic diseases: type 2 diabetes, cardiovascular disease, metabolic syndrome, nonalcoholic fatty liver disease, and degenerative joint disease. Importantly, however, the development of these chronic conditions is gradual, with opportunities for interventions, especially at an early stage, while physiological and metabolic abnormalities are reversible. While restoration of health is feasible, it is nevertheless challenging when addressed in an incomplete way that does not include attention to psychological as well as nutritional and physical components.

## 4 INTEGRATED LIFESTYLE INTERVENTIONS TO SUPPORT HEALTHY AGING

The World Health Organization has developed guidance emphasizing the role of community and primary care providers in delivering integrated lifestyle and drug therapies to optimize healthy aging (World Health Organization, 2019). A major challenge, however, is that lifestyle interventions require support for long‐term behavior change to occur. Such support can include health coaching, social work, physical therapy, and nutrition counseling, ideally combined and continued as needed throughout the individual's life. In the United States, these services are frequently covered for a limited time by insurance plans or not covered at all, which creates unequal access for those without the ability to pay out of pocket. Clinical trials that evaluate lifestyle interventions in a way that assesses and ensures equitable access to whole‐person care are needed to build the evidence base. In this type of clinical research, it may be advantageous to measure the effect of a “bundled” intervention including multiple components, as is being increasingly done in other fields, such as integrative pain care (Seal et al., 2020). This type of “pragmatic” trial is increasingly viewed as advantageous to understanding the effectiveness of treatments in the “real world.” In these types of trials, the study is designed to test the effectiveness of the bundled intervention compared with usual care, rather than a factorial design that would be aimed at determining which of the components of the bundle is responsible for any therapeutic effects.

An important challenge in building clinical research evidence on healthy aging is the choice and number of outcome measures. Unlike drugs, lifestyle modification, such as beginning an exercise program, has effects on multiple systems that, together, could contribute to improving the health of the whole person. Although identifying a primary outcome is important in any clinical trial, measuring multiple secondary outcomes to examine “whole person” effects can be done, as long as appropriate statistical adjustments are applied to the analysis (Parker & Weir, 2022). Furthermore, the framework of whole‐person health encourages the use of advanced statistical approaches and multiscale modeling techniques that are now available (National Center for Complementary and Integrative Health, 2021b). For example, longitudinal network analyses on large datasets can identify patterns of change within the network over time, either in longitudinal cohorts where lifestyle behaviors are tracked or in randomized trials of lifestyle interventions that include measurements at several time points. This type of network analysis is based on measuring the changing relationships between multiple outcomes rather than testing them one at a time (Borsboom et al., 2021) and could be applied to understand the multisystem nature of healthy aging.

Throughout the world, traditional systems of care, many of which have been used for millennia, share common attributes, including attention to the whole person. The field of complementary and integrative health includes multicomponent therapeutic systems, such as traditional Chinese medicine and Ayurveda, that incorporate nutritional, physical, and psychological components as part of the treatment framework. Despite their widespread use, research on “whole health systems,” including their effects on aging, is still in its infancy, in part due to challenges posed by the inherent complexity of these systems of care. Another hurdle in conducting research testing the efficacy and mechanisms of traditional whole health systems is that they typically use a theoretical and diagnostic framework that is different from that of conventional medicine, which focuses on individual organs and systems (Shea, 2006). An important step toward conducting large‐scale, rigorous studies of these diagnostic criteria is establishing standardized protocols and testing their reproducibility across different research settings. These interventions may involve mechanisms that we have not yet explored or are just beginning to explore, such as modulation of autonomic nervous system activity with acupuncture (Napadow et al., 2013). As mechanisms become better understood, objective physiological measures can be incorporated into clinical trials in addition to patient‐reported outcomes. Eventually, including a plurality of perspectives in rigorous research, in addition to Western medicine, will enrich our understanding of health overall.

## 5 FUTURE DIRECTIONS FOR RESEARCH ON WHOLE‐PERSON CARE FOR HEALTHY AGING

An important challenge to be overcome is to demonstrate whether an intervention used in early life or middle age can lead to healthy aging several decades later. This requires a combination of long‐term observational studies (Quach et al., 2017) as well as randomized trials of interventions with very long time horizons, such as the Physicians' Health Study (2012), and it becomes increasingly challenging when the intervention has multiple components. A further challenge is to determine the cost‐effectiveness of integrated lifestyle interventions, which may require more substantial cost “investment” in early and mid‐life, compared with pharmacologic treatments. Determining whether these interventions are cost‐effective in the long term, that is, over the course of a lifetime, requires long‐term follow‐up over several decades, which is beyond the scope of most clinical trials and funding mechanisms. Embedding randomized trials within established longitudinal cohorts, such as the National Institutes of Health (NIH) All of Us Research Program (All of Us Research Program, 2023), and using cutting‐edge network analysis methods offer the possibility of testing the effects of lifestyle modification starting early in life over the course of the lifespan.

In conclusion, research is needed to determine the long‐term effectiveness, cost‐effectiveness, and mechanisms of action of integrated lifestyle interventions in the promotion of healthy aging. A recent report by the National Academies of Sciences, Engineering, and Medicine (2023) has highlighted the importance of a whole‐person approach in the future of health care. NCCIH's current strategic plan has embraced the ambitious but vital goal of understanding how to best support healthy aging in the context of the whole person, calling for rigorous research applications to study multicomponent interventions, including medications when indicated for established disease. With continued research and development, this work will potentially support a more holistic approach to care that addresses the complex interplay of biological, psychological, social, and environmental factors that impact health and well‐being across the lifespan.

## AUTHOR CONTRIBUTIONS

All authors made substantial contributions to the conception, design, and drafting of this manuscript.

## FUNDING INFORMATION

No funding information provided.

## CONFLICT OF INTEREST STATEMENT

The authors have no conflicts of interest to declare.

## Data Availability

Data sharing not applicable to this article as no datasets were generated or analyzed during the current study.
